# Curve intersection based on cubic hybrid clipping

**DOI:** 10.1186/s42492-022-00114-3

**Published:** 2022-06-22

**Authors:** Yaqiong Wu, Xin Li

**Affiliations:** School of Mathematical Science, University of Science and Technology of China, Hefei, 200026 Anhui China

**Keywords:** Bézier curve, Curve intersection, Hybrid clipping

## Abstract

This study presents a novel approach to computing all intersections between two Bézier curves using cubic hybrid clipping. Each intersection is represented by two strip intervals that contain an intersection. In each step, one curve is bounded by two fat lines, and the other is bounded by two cubic Bézier curves, clipping away the domain that does not contain the intersections. By selecting the moving control points of the cubic hybrid curves, better cubic polynomial bounds are obtained to make the proposed method more efficient. It was proved that the two strip intervals have second- and fourth-order convergence rates for transversal intersections. Experimental results show that the new algorithm is the most efficient among all existing curve/curve intersection approaches.

## Introduction

Given an interval [*α*, *β*] ⊂ *ℝ*, a Bézier curve **P**(*t*), *t* ∈ [*α*, *β*] is defined as

1 $$\mathbf{P}(t)=\sum_{i=0}^n{\mathbf{P}}_i{B}_{i,\left[\alpha, \beta \right]}^n(t)$$

where ${B}_{i,\left[\alpha, \beta \right]}^n(t)=\left(\genfrac{}{}{0pt}{}{n}{i}\right)\frac{{\left(t-\alpha \right)}^i{\left(\beta -t\right)}^{n-i}}{{\left(\beta -\alpha \right)}^n},i=0,1,\dots, n$ are Bernstein polynomials in [*α*, *β*], and ${\left\{{\mathbf{P}}_i\right\}}_{i=0}^n$ are the control points [1]. Given two Bézier curves **P**(*t*), *t* ∈ [*α*, *β*] and **Q**(*s*), *s* ∈ [*ξ*, *η*], the problem considered in the present study is how to compute all (*t*^∗^, *s*^∗^), *t*^∗^ ∈ [*α*, *β*], *s*^∗^ ∈ [*ξ*, *η*] such that **P**(*t*^∗^) = **Q**(*s*^∗^). Various methods have been developed to solve this task, such as a subdivision-based approach [2], binary subdivision approach [3], implicitization [4] and Bézier clipping [5].

The most common approach consists of clipping away the regions of the curves that are guaranteed to not intersect. Each intersection parameter pair (*s*^∗^, *t*^∗^) is replaced with an interval that is iteratively computed. The *k*-th iteration interval is denoted as [*α*_*k*_, *β*_*k*_] × [*ξ*_*k*_, *η*_*k*_]. Let *h*_*k*_ = *β*_*k*_ − *α*_*k*_ and *d*_*k*_ = *η*_*k*_ − *ξ*_*k*_. If a constant *γ*_*i*_ exists such that

2 $${h}_{k+1}\le {C}_1{h}_k^{\gamma_1}+{C}_2{d}_k^{\gamma_2},{d}_{k+1}\le {C}_3{h}_k^{\gamma_3}+{C}_4{d}_k^{\gamma_4}$$

where *C*_*i*_ are constants independent of *k* and the curves, and thus *γ*_*i*_ is the convergence rate of the sequence intervals {[*α*_*k*_, *β*_*k*_]}_*k*_ and {[*ξ*_*k*_, *η*_*k*_]}_*k*_. The key problem is to find an algorithm for which *γ*_*i*_ is as large as possible with as few computations as possible during each iteration.

The above problem plays an important role in many engineering fields, such as computer-aided design and manufacturing (CAD/CAM), collision detection, and geometric modeling [1], and is a basic operation in solid modeling. In geometric processing, the intersections and intersection curves in a solid model are extremely important for the visualization, analysis, and manufacturing of the model [6]. With the continuous development of computer-aided geometric design and CAD/CAM, as well as the continuous progress made in science and technology, the numbers of calculations and data to be processed for intersection problems are increasing. It is therefore important to develop efficient and stable methods for dealing with intersection problems.

To solve such problems, the Bézier clipping algorithm introduced in ref. [5] is a widely used, fast, and robust method. The Bézier clipping algorithm was proven to have a second-order convergence rate [7]. Subsequently, several different approaches have been proposed to improve the Bézier clipping algorithm. Bartoň and Jüttler [8] and Liu et al. [9] developed quadratic and cubic clipping techniques based on a degree reduction to compute all roots of a univariate polynomial equation. Lou and Liu [10] extended the approach in ref. [9] to curve/curve intersection problems and proved that the algorithm achieves at least a second-order convergence rate. In addition, North [11] developed a geometry interval clipping algorithm based on quadratic hybrid curves [12] for use with curve/curve intersection problems, Liu and Li [13] proved that the algorithm achieves a quadratic convergence rate. Moreover, Yuan [14] recently developed a cubic hybrid clipping (HybClip) based on hybrid curves to compute all roots of a univariate polynomial equation with a numerically verified fourth-order convergence rate.

In this study, the approach in ref. [14] is extended to handle curve/curve intersection problems. Unlike the approach in ref. [14], a better bound is chosen for cubic HybClip, and thus the algorithm requires 8% less time than a method that directly uses the cubic hybrid curve [14]. In addition, it is proved that the two sequences in the new clipping algorithm have second- and fourth-order convergence rates. Subsequently, a complete comparison is provided with all existing curve/curve intersection algorithms based on subdivisions on a random 40,000 curve/curve intersection database. The new algorithm requires 30% less time than the geometry interval clipping algorithm [11] and 60% less time than the cubic clipping algorithm [10].

The remainder of this paper is organized as follows. In Methods section, the cubic hybrid curves are presented with two moving control points, and the details of the curve/curve intersection algorithms are described when applied in both 2D and 3D. In Results section, a proof of the convergence rate of the new intersection algorithm is provided, and the six techniques are compared from various perspectives. Finally, some concluding remarks are provided in Conclusions section and areas of future work are discussed in Discussion section.

## Methods

### Hybrid curve

**Fig. 1:**
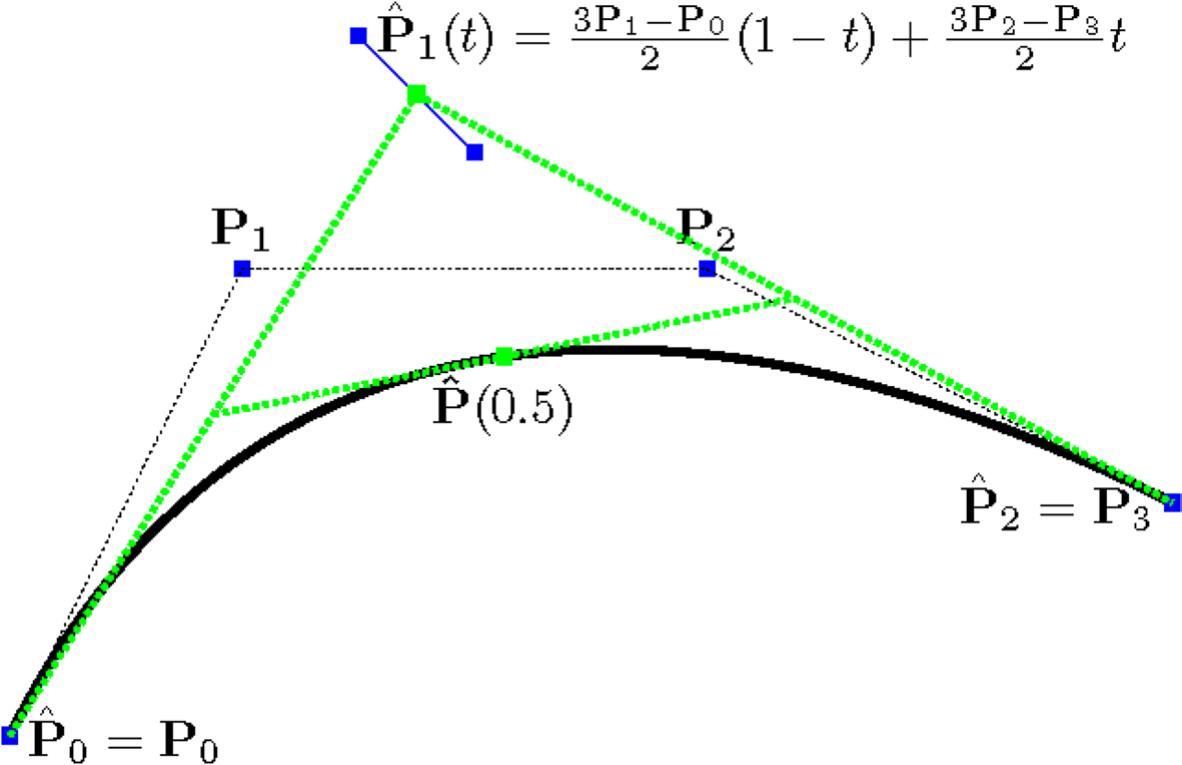
A cubic Bézier curve expressed as a quadratic hybrid curve

A hybrid curve refers to a curve with at least one moving control point, which is itself a parametric curve and shares one parameter with the hybrid. Sederberg and Kakimoto [12] originated the idea of using hybrid polynomial Bézier curves to approximate rational Bézier curves. Later, North [11] transformed all polynomial Bézier curves of degree *d* ≥ 2 into equivalent quadratic hybrid curves with a single moving control point and fixed endpoints. As an illustration, a simple quadratic hybrid curve was constructed with a single moving control point, equivalent to a cubic Bézier curve, as shown in Fig. 1.

3 $${\displaystyle \begin{array}{ll}\mathbf{P}(t)& ={\mathbf{P}}_0{\left(1-t\right)}^3+3{\mathbf{P}}_1{\left(1-t\right)}^2t+3{\mathbf{P}}_2\left(1-t\right){t}^2+{\mathbf{P}}_3{t}^3\\ {}& ={\mathbf{P}}_0{B}_0^2(t)+\left(\frac{3{\mathbf{P}}_1-{\mathbf{P}}_0}{2}\left(1-t\right)+\frac{3{\mathbf{P}}_2-{\mathbf{P}}_3}{2}t\right){B}_1^2(t)+{\mathbf{P}}_3{B}_2^2(t)\end{array}}$$

To evaluate a point on a hybrid curve, the locations of all moving control points are first determined at the given parameter value *t*. Once the moving control points are determined, the hybrid curve can be evaluated as a common curve. For example, to evaluate $\hat{\mathbf{P}}(t)$ at *t* = 0.5, ${\hat{\mathbf{P}}}_1(0.5)$ is first evaluated and the resulting point is then used to evaluate $\hat{\mathbf{P}}(0.5)$, as shown in Fig. 1.

Using the same principles as in ref. [11], by properly selecting the moving control points, the hybrid curve can produce any traditional Bézier curve. In this study, a hybrid curve having the following form is focused on:

**Theorem 1. ***Given a degree n* ≥ 3 *Bézier curve ***P**(*t*) *with control points *${\left\{{\mathbf{P}}_i\right\}}_{i=0}^n$*, there exists an equivalent cubic hybrid curve *$\hat{\mathbf{P}}(t)$* with two fixed control points *${\hat{\mathbf{P}}}_0={\mathbf{P}}_0,{\hat{\mathbf{P}}}_3={\mathbf{P}}_n$* and two moving control points *${\hat{\mathbf{P}}}_1(t),{\hat{\mathbf{P}}}_2(t)$*. The two moving control points *${\hat{\mathbf{P}}}_1(t),{\hat{\mathbf{P}}}_2(t)$* are Bézier curves of degree n* − 3 *with control points *${\left\{{\hat{\mathbf{P}}}_{1,i-1}\right\}}_{i=1}^{n-2}$* and *${\left\{{\hat{\mathbf{P}}}_{2,i-2}\right\}}_{i=2}^{n-1}$*, respectively, where*

4 $$\frac{n-i-1}{n-2}{\hat{\mathbf{P}}}_{1,i-1}+\frac{i-1}{n-2}{\hat{\mathbf{P}}}_{2,i-2}=\frac{a_i{\mathbf{P}}_0+{b}_i{\mathbf{P}}_i+{c}_i{\mathbf{P}}_n}{a_i+{b}_i+{c}_i}$$

*a*_*i*_ =  − (*n* − *i*)(*n* − *i* − 1)(*n* − *i* − 2), *b*_*i*_ = *n*(*n* − 1)(*n* − 2), *c*_*i*_ =  − *i*(*i* − 1)(*i* − 2), and *i* ∈ {1, …, *n* − 1}.

*Proof. *The degree *n* × *m* tensor product Bézier surface patch [1] is defined as

5 $$\mathbf{Q}\left(s,t\right)=\sum_{i=0}^n\sum_{j=0}^m{\mathbf{Q}}_{i,j}{B}_i^n(s){B}_j^m(t)$$

where ${B}_i^n(s){B}_j^m(t),0\le s,t\le 1$ is the product of the two Bernstein bases in [0, 1], and **Q**_*i*, *j*_, *i* = 0, …, *n*; *j* = 0, …, *m* are the control points of **Q**(*s*, *t*).

From ref. [15], a degree *m* + *d* Bézier curve **P**(*t*) with control points **P**_*i*_ can be described as the diagonal curve **P**(*t*) = **Q**(*t*, *t*) of a degree *m* × *d* Bézier surface **Q**(*s*, *t*), i.e.,

6 $${\mathbf{P}}_i=\frac{1}{\left(\genfrac{}{}{0pt}{}{m+d}{i}\right)}\sum_{j+k=i}\left(\genfrac{}{}{0pt}{}{m}{j}\right)\left(\genfrac{}{}{0pt}{}{d}{k}\right){\mathbf{Q}}_{j,k}$$

If *m* = 3, then *j* ∈ {0, 1, 2, 3} and (*j*, *k*) ∈ {(0, *i*), (1, *i* − 1), (2, *i* − 2), (3, *i* − 3)}. Expanding the summation and rearranging the terms, the following is obtained:

7 $${\displaystyle \begin{array}{ll}\left(\genfrac{}{}{0pt}{}{d+3}{i}\right){\mathbf{P}}_i& =\left(\genfrac{}{}{0pt}{}{3}{0}\right)\left(\genfrac{}{}{0pt}{}{d}{i}\right){\mathbf{Q}}_{0,i}+\left(\genfrac{}{}{0pt}{}{3}{1}\right)\left(\genfrac{}{}{0pt}{}{d}{i-1}\right){\mathbf{Q}}_{1,i-1}\\ {}& +\left(\genfrac{}{}{0pt}{}{3}{2}\right)\left(\genfrac{}{}{0pt}{}{d}{i-2}\right){\mathbf{Q}}_{2,i-2}+\left(\genfrac{}{}{0pt}{}{3}{3}\right)\left(\genfrac{}{}{0pt}{}{d}{i-3}\right){\mathbf{Q}}_{3,i-3}\end{array}}$$

If the control points **P**_*i*_ of degree *n* = *d* + 3 diagonal curve **P**(*t*) are known, **Q**_0, *i*_ = **P**_0_ and **Q**_3, *i* − 3_ = **P**_*n*_ can be set. Thus,

8 $${\displaystyle \begin{array}{ll}& \left(\genfrac{}{}{0pt}{}{n}{i}\right){\mathbf{P}}_i=\left(\genfrac{}{}{0pt}{}{n-3}{i}\right){\mathbf{P}}_0+3\left(\genfrac{}{}{0pt}{}{n-3}{i-1}\right){\mathbf{Q}}_{1,i-1}+3\left(\genfrac{}{}{0pt}{}{n-3}{i-2}\right){\mathbf{Q}}_{2,i-2}+\left(\genfrac{}{}{0pt}{}{n-3}{i-3}\right){\mathbf{P}}_n\\ {}& \left(\genfrac{}{}{0pt}{}{n-3}{i-1}\right){\mathbf{Q}}_{1,i-1}+\left(\genfrac{}{}{0pt}{}{n-3}{i-2}\right){\mathbf{Q}}_{2,i-2}=\frac{1}{3}\left[\left(\genfrac{}{}{0pt}{}{n}{i}\right){\mathbf{P}}_i-\left(\genfrac{}{}{0pt}{}{n-3}{i}\right){\mathbf{P}}_0-\left(\genfrac{}{}{0pt}{}{n-3}{i-3}\right){\mathbf{P}}_n\right]\end{array}}$$

Simplifying the above formulas, the following is achieved:

9 $${\displaystyle \begin{array}{ll}& \frac{n-i-1}{n-2}{\mathbf{Q}}_{1,i-1}+\frac{i-1}{n-2}{\mathbf{Q}}_{2,i-2}=\\ {}& \frac{\left(i-n\right)\left(n-i-1\right)\left(n-i-2\right)}{3i\left(n-i\right)\left(n-2\right)}{\mathbf{P}}_0+\frac{n\left(n-1\right)\left(n-2\right)}{3i\left(n-i\right)\left(n-2\right)}{\mathbf{P}}_i+\frac{-i\left(i-1\right)\left(i-2\right)}{3i\left(n-i\right)\left(n-2\right)}{\mathbf{P}}_n\end{array}}$$

for *i* ∈ {1, …, *n* − 1}. Setting *a*_*i*_ =  − (*n* − *i*)(*n* − *i* − 1)(*n* − *i* − 2), *b*_*i*_ = *n*(*n* − 1)(*n* − 2), *c*_*i*_ =  − *i*(*i* − 1)(*i* − 2), and observing that *a*_*i*_ + *b*_*i*_ + *c*_*i*_ = 3*i*(*n* − *i*)(*n* − 2), the following occur:

10 $$\frac{n-i-1}{n-2}{\mathbf{Q}}_{1,i-1}+\frac{i-1}{n-2}{\mathbf{Q}}_{2,i-2}=\frac{a_i{\mathbf{P}}_0+{b}_i{\mathbf{P}}_i+{c}_i{\mathbf{P}}_n}{a_i+{b}_i+{c}_i}$$

Because **Q**_0, *i*_ = **P**_0_ and **Q**_3, *i* − 3_ = **P**_*n*_, the *s* = *t* diagonal curve of **Q**(*s*, *t*) can be evaluated using the following formula:

11 $$\mathbf{Q}\left(t,t\right)={\left(1-t\right)}^3{\mathbf{P}}_0+3{\left(1-t\right)}^2t{\hat{\mathbf{P}}}_1(t)+3\left(1-t\right){t}^2{\hat{\mathbf{P}}}_2(t)+{t}^3{\mathbf{P}}_n$$

where ${\hat{\mathbf{P}}}_1(t)\ \mathrm{and}{\hat{\ \mathbf{P}}}_2(t)$ are the degree *n* − 3 Bézier curves comprising the control points ${\hat{\mathbf{P}}}_{1,i-1}$ and ${\hat{\mathbf{P}}}_{2,i-2}$, respectively, where

12 $${\hat{\mathbf{P}}}_{1,i-1}={\mathbf{Q}}_{1,i-1},i=1,\dots, n-2\kern1em \mathrm{and}\kern1em {\hat{\mathbf{P}}}_{2,i-2}={\mathbf{Q}}_{2,i-2},i=2,\dots, n-1$$

This is a cubic hybrid curve with two moving control points ${\hat{\mathbf{P}}}_1(t),{\hat{\mathbf{P}}}_2(t)$, and fixed control points **P**_0_, **P**_*n*_.

From Theorem 1, if *i* = 1 or *i* = *n* − 1, the first control point of ${\hat{\mathbf{P}}}_1(t)$ and the last control point of ${\hat{\mathbf{P}}}_2(t)$ are fixed as follows:

13 $${\hat{\mathbf{P}}}_{1,0}=\frac{\left(3-n\right){\mathbf{P}}_0+n{\mathbf{P}}_1}{3}\kern1em \mathrm{and}\kern1em {\hat{\mathbf{P}}}_{2,n-3}=\frac{\left(3-n\right){\mathbf{P}}_n+n{\mathbf{P}}_{n-1}}{3}$$

Theorem 1 indicates that the two moving control points are relevant, for which three cases are discussed:

#### Case 1

If the first moving control point is a fixed point denoted by ${\hat{\mathbf{Q}}}_1$ and the second moving control point is denoted by ${\hat{\mathbf{Q}}}_2(t)$, then the control points ${\left\{{\hat{\mathbf{Q}}}_{2,i}\right\}}_{i=0}^{n-3}$ of ${\hat{\mathbf{Q}}}_2(t)$ can be calculated from Eq. (4), as indicated by Yuan [14]:

14 $$\hat{\mathbf{Q}}(t)={\mathbf{P}}_0{B}_0^3(t)+{\hat{\mathbf{Q}}}_1{B}_1^3(t)+{\hat{\mathbf{Q}}}_2(t){B}_2^3(t)+{\mathbf{P}}_n{B}_3^3(t)$$

#### Case 2

If the second moving control point is a fixed point denoted by ${\hat{\mathbf{R}}}_2$ and the first moving control point is denoted by ${\hat{\mathbf{R}}}_1(t)$, the control points ${\left\{{\hat{\mathbf{R}}}_{1,i}\right\}}_{i=0}^{n-3}$ of ${\hat{\mathbf{R}}}_1(t)$ can be obtained from Eq. (4), i.e.,

15 $$\hat{\mathbf{R}}(t)={\mathbf{P}}_0{B}_0^3(t)+{\hat{\mathbf{R}}}_1(t){B}_1^3(t)+{\hat{\mathbf{R}}}_2{B}_2^3(t)+{\mathbf{P}}_n{B}_3^3(t)$$

#### Case 3

In general, ${\hat{\mathbf{P}}}_1(t)$ and ${\hat{\mathbf{P}}}_2(t)$ are moving to control points. Because they are equivalent to **P**(*t*),

16 $${\displaystyle \begin{array}{rrrr}\mathbf{P}(t)& =\hat{\mathbf{Q}}(t)& =& {\mathbf{P}}_0{B}_0^3(t)+{\hat{\mathbf{Q}}}_1{B}_1^3(t)+{\hat{\mathbf{Q}}}_2(t){B}_2^3(t)+{\mathbf{P}}_n{B}_3^3(t)\\ {}& =\hat{\mathbf{R}}(t)& =& {\mathbf{P}}_0{B}_0^3(t)+{\hat{\mathbf{R}}}_1(t){B}_1^3(t)+{\hat{\mathbf{R}}}_2{B}_2^3(t)+{\mathbf{P}}_n{B}_3^3(t)\\ {}& =\hat{\mathbf{P}}(t)& =& {\mathbf{P}}_0{B}_0^3(t)+{\hat{\mathbf{P}}}_1(t){B}_1^3(t)+{\hat{\mathbf{P}}}_2(t){B}_2^3(t)+{\mathbf{P}}_n{B}_3^3(t)\end{array}}$$

Hence, *λ* ∈ [0, 1] exists such that

17 $$\left(1-\lambda \right)\hat{\mathbf{Q}}(t)+\lambda \hat{\mathbf{R}}(t)=\hat{\mathbf{P}}(t)$$

Through a simple approach,

18 $$\left(1-\lambda \right){\hat{\mathbf{Q}}}_1+\lambda {\hat{\mathbf{R}}}_1(t)={\hat{\mathbf{P}}}_1(t)\kern1em \mathrm{and}\kern1em \left(1-\lambda \right){\hat{\mathbf{Q}}}_2(t)+\lambda {\hat{\mathbf{R}}}_2={\hat{\mathbf{P}}}_2(t)$$

and obtain

19 $${\hat{\mathbf{P}}}_{1,i}=\left(1-\lambda \right){\hat{\mathbf{Q}}}_1+\lambda {\hat{\mathbf{R}}}_{1,i}\kern1em \mathrm{and}\kern1em {\hat{\mathbf{P}}}_{2,i}=\left(1-\lambda \right){\hat{\mathbf{Q}}}_{2,i}+\lambda {\hat{\mathbf{R}}}_2$$

where *i* ∈ {0, …, *n* − 3}; in addition, ${\hat{\mathbf{Q}}}_1,{\hat{\mathbf{Q}}}_{2,i}$ and ${\hat{\mathbf{R}}}_{1,i},{\hat{\mathbf{R}}}_2$ are known from the two cases above, and based on Eqs. (4) and (13), the two moving control points depend on the value of *λ*.

### Curve/curve intersection based on cubic HybClip

Given two Bézier curves **P**(*t*), *t* ∈ [*α*, *β*] and **Q**(*s*), *s* ∈ [*ξ*, *η*], in this section, a cubic hybrid clipping algorithm is proposed for computing all intersections.

#### 2D curve/curve intersection

**Fig. 2:**
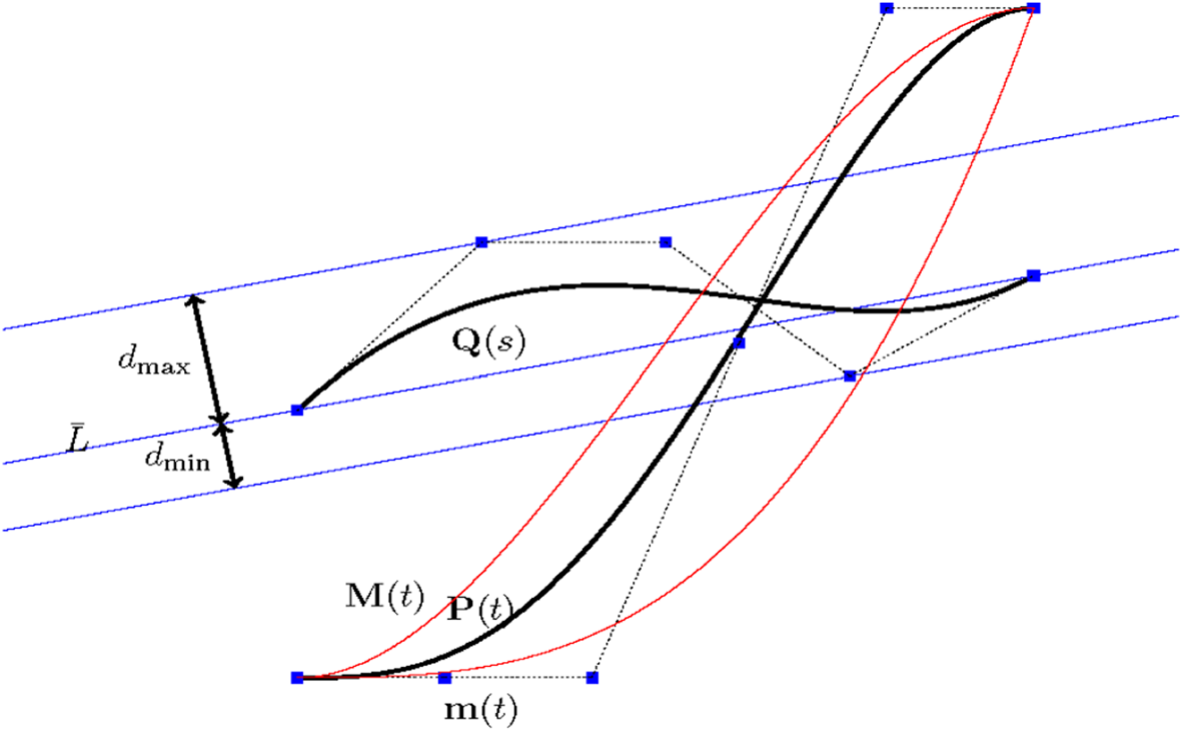
Intersection of cubic strip [***m***, ***M***] of ***P*** and fat line ***L***_***Q***_ of ***Q***

The algorithm for two planar Bézier curves is first discussed. This algorithm is presented in Algorithm 1, and illustrated in Fig. 2.

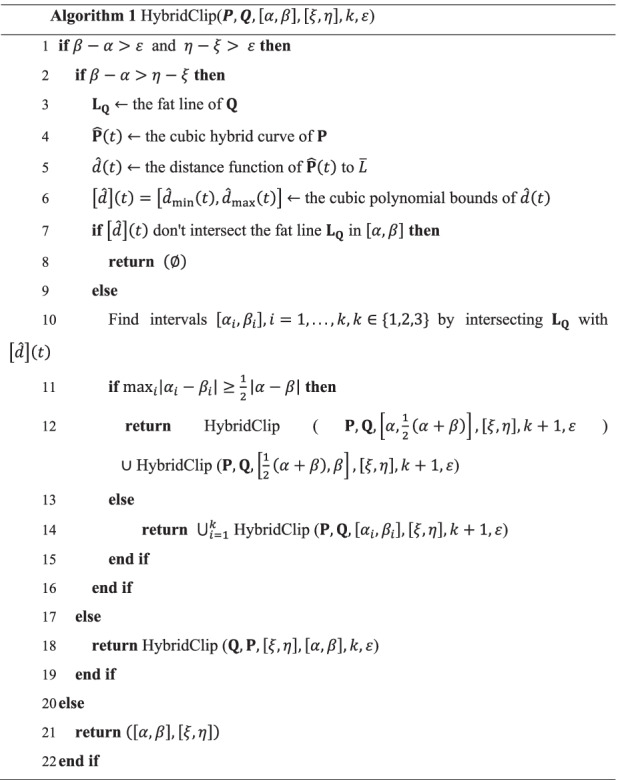



In each step, one curve is bounded by two lines, called fat lines, which were first introduced in ref. [5]. Let $\overline{L}$ be a line passing through **P**_0_ and **P**_*n*_ of a degree *n* Bézier curve, **P**(*t*), and suppose $\overline{L}$ has an implicit equation:

20 $$d\left(x,y\right)= ax+ by+c=0,\left({a}^2+{b}^2=1\right)$$

The fat line of **P** is defined as a region

21 $${\mathbf{L}}_{\mathbf{P}}=\left\{\left(x,y\right)|d\left(x,y\right)\in \left[{d}_{\mathrm{min}},{d}_{\mathrm{max}}\right]\right\}$$

where [*d*_min_, *d*_max_] = [min_0 ≤ *i* ≤ *n*_*d*(**P**_*i*_), max_0 ≤ *i* ≤ *n*_*d*(**P**_*i*_)], and *d*(**P**_*i*_) = *ax*_*i*_ + *by*_*i*_ + *c*, **P**_*i*_ = (*x*_*i*_, *y*_*i*_).

The steps of Algorithm 1 are described in more detail in the following:

1. In line 1, if the intervals are within the specified level of accuracy, the parameter intervals corresponding to the intersection in line 21 can be directly obtained.
2. In line 2, the curve with a larger priority parameter interval is always clipped.
3. In line 5, $\hat{d}(t)$ is a cubic hybrid polynomial in the Bernstein basis,

22 $$\hat{d}(t)=d\left(\hat{\mathbf{P}}(t)\right)={\left(1-t\right)}^3{\hat{d}}_0+3t{\left(1-t\right)}^2{\hat{d}}_1(t)+3{t}^2\left(1-t\right){\hat{d}}_2(t)+{t}^3{\hat{d}}_3$$

where

$${\displaystyle \begin{array}{ll}& i=0,3,{\hat{d}}_i=d\left({\hat{\mathbf{P}}}_i\right)\\ {}& i=1,2,{\hat{d}}_i(t)=d\left({\hat{\mathbf{P}}}_i(t)\right)=\sum_{j=0}^{n-3}d\left({\hat{\mathbf{P}}}_{i,j}\right){B}_j^{n-3}(t)\end{array}}$$

(4) In line 6, to obtain the cubic lower and upper bounds, defining $\left[{\hat{d}}_1\right],\left[{\hat{d}}_2\right]$ as the intervals containing the coefficients $d\left({\hat{\mathbf{P}}}_{1,j}\right)$ and $d\left({\hat{\mathbf{P}}}_{2,j}\right)$ of ${\hat{d}}_1(t),{\hat{d}}_2(t)$, respectively, $\hat{d}(t)$ is bound using an interval Bernstein polynomial [16]:

23 $${\displaystyle \begin{array}{ll}& \left[\hat{d}\right](t)={B}_0^3(t){\hat{d}}_0+{B}_1^3(t)\left[{\hat{d}}_1\right]+{B}_2^3(t)\left[{\hat{d}}_2\right]+{B}_3^3(t){\hat{d}}_3\\ {}& \left[{\hat{d}}_i\right]=\left[{\hat{d}}_{i,\min },{\hat{d}}_{i,\max}\right]=\left[\underset{0\le j\le n-3}{\min }d\left({\hat{\mathbf{P}}}_{i,j}\right),\underset{0\le j\le n-3}{\max }d\left({\hat{\mathbf{P}}}_{i,j}\right)\right]\end{array}}$$

The lower and upper bounds of $\left[\hat{d}\right](t)$ are defined through cubic polynomials in a simple manner:

24 $${\displaystyle \begin{array}{ll}& {\hat{d}}_{\mathrm{min}}(t)={B}_0^3(t){\hat{d}}_0+{B}_1^3(t){\hat{d}}_{1,\min }+{B}_2^3(t){\hat{d}}_{2,\min }+{B}_3^3(t){\hat{d}}_3\\ {}& {\hat{d}}_{\mathrm{max}}(t)={B}_0^3(t){\hat{d}}_0+{B}_1^3(t){\hat{d}}_{1,\max }+{B}_2^3(t){\hat{d}}_{2,\max }+{B}_3^3(t){\hat{d}}_3\end{array}}$$

Hence, ${\hat{d}}_{\mathrm{min}}(t)\le \hat{d}(t)\le {\hat{d}}_{\mathrm{max}}(t)$. To obtain a tighter bound $\left[\hat{d}\right](t)$, the following optimization function is used:

25 $${\displaystyle \begin{array}{ll}& \min \left\{{\hat{d}}_{\mathrm{max}}(t)-{\hat{d}}_{\mathrm{min}}(t)\right\}\\ {}& =\min \left\{{\hat{d}}_{1,\max }-{\hat{d}}_{1,\min }+{\hat{d}}_{2,\max }-{\hat{d}}_{2,\min}\right\}\\ {}& =\min \left\{\underset{j}{\max}\left({\hat{d}}_{1,j}\right)-\underset{j}{\min}\left({\hat{d}}_{1,j}\right)+\underset{j}{\max}\left({d}_{2,j}\right)-\underset{j}{\min}\left({d}_{2,j}\right)\right\}\end{array}}$$

where ${\hat{d}}_{i,j}=d\left({\hat{\mathbf{P}}}_{i,j}\right),i=1,2$, and *j* ∈ {0, 1, …, *n* − 3}. In Eq. (22), if the second moving point of $\hat{d}(t)$ is a fixed point denoted by ${\hat{q}}_2$, and the first moving point is denoted by ${\hat{q}}_1(t)$, the following is obtained:

26 $$\hat{q}(t)={B}_0^3(t){\hat{d}}_0+{B}_1^3(t){\hat{q}}_1(t)+{B}_2^3(t){\hat{q}}_2+{B}_3^3(t){\hat{d}}_3$$

If the first moving point of $\hat{d}(t)$ is a fixed point denoted by ${\hat{r}}_1$, and the second moving point is denoted by ${\hat{r}}_2(t)$, the following is obtained:

27 $$\hat{r}(t)={B}_0^3(t){\hat{d}}_0+{B}_1^3(t){\hat{r}}_1+{B}_2^3(t){\hat{r}}_2(t)+{B}_3^3(t){\hat{d}}_3$$

There exists *λ* ∈ [0, 1] such that

28 $$\left(1-\lambda \right)\hat{q}(t)+\lambda \hat{r}(t)=\hat{d}(t)$$

From Eqs. (19) and (25), the problem becomes linear, i.e.,

29 $${\displaystyle \begin{array}{ll}& \min \left\{{\hat{d}}_{\mathrm{max}}(t)-{\hat{d}}_{\mathrm{min}}(t)\right\}\\ {}& =\left(1-\lambda \right)\left(\underset{j}{\max}\left({\hat{q}}_{1,j}\right)-\underset{j}{\min}\left({\hat{q}}_{1,j}\right)\right)+\lambda \left(\underset{j}{\max}\left({\hat{r}}_{2,j}\right)-\underset{j}{\min}\left({\hat{r}}_{2,j}\right)\right)\end{array}}$$

where *j* ∈ {0, 1, …, *n* − 3}. Let $$={\max}_j\left({\hat{q}}_{1,j}\right)-{\min}_j\left({\hat{q}}_{1,j}\right)\ \mathrm{and}$$$$b={\max}_j\left({\hat{r}}_{2,j}\right)-{\min}_j\left({\hat{r}}_{2,j}\right)$$. If *a* ≥ *b*, *λ* = 1 is set in Eq. (28). Otherwise, *λ* = 0. The tighter cubic bounds of $$\hat{d}(t)$$ can then be obtained using Eqs. (23) and (24).

**Fig. 3:**
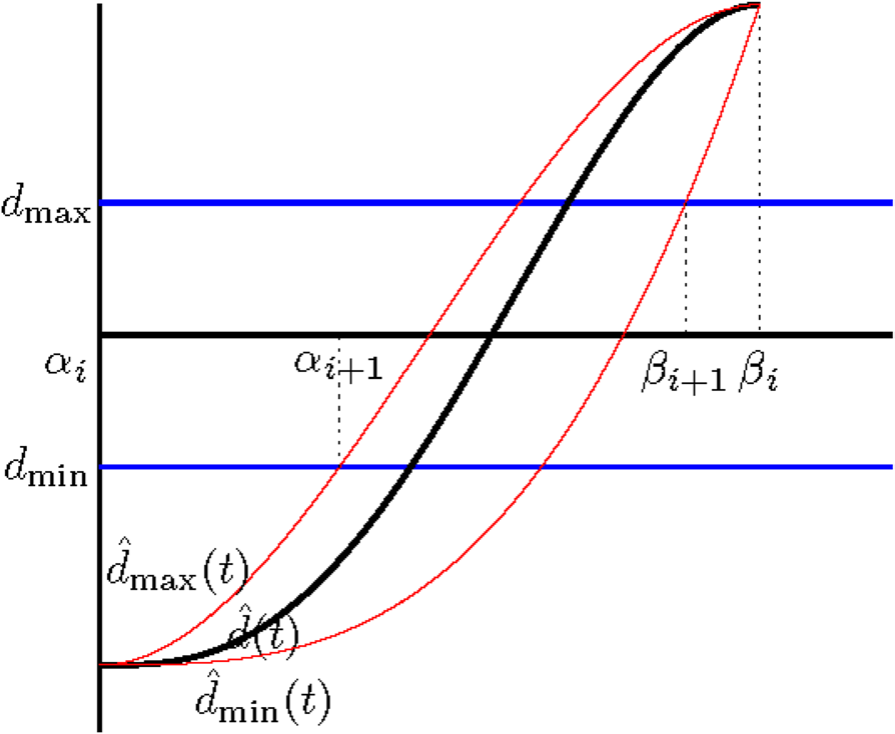
Clipping computed from $\left[\hat{d}\right](t)\cap \left[{d}_{min},{d}_{max}\right]=\varnothing$

(5) In lines 7–10, the intervals of *t* for which **P**(*t*) lies outside of **L**_**Q**_ correspond to regions where $\left[\hat{d}\right](t)\cap \left[{d}_{\mathrm{min}},{d}_{\mathrm{max}}\right]=\varnothing$, as shown in Fig. 3. The values *t* ∈ [0, 1], for which ${\hat{d}}_{\mathrm{min}}(t)$ and ${\hat{d}}_{\mathrm{max}}(t)$ cross *d*_min_ and *d*_max_, correspond to the roots of

30 $${\displaystyle \begin{array}{rrr}& {\hat{d}}_{\mathrm{min}}(t)={d}_{\mathrm{min}},& {\hat{d}}_{\mathrm{min}}(t)={d}_{\mathrm{max}}\\ {}& {\hat{d}}_{\mathrm{max}}(t)={d}_{\mathrm{min}},& {\hat{d}}_{\mathrm{max}}(t)={d}_{\mathrm{max}}\end{array}}$$

Because ${\hat{d}}_{\mathrm{min}}(t)$ and ${\hat{d}}_{\mathrm{max}}(t)$ are cubic polynomials, these roots can be solved directly using the cubic formula.

(6) In line 7, if the intersection is empty, no intersection exists between the two curves. In line 11, if the lengths of these intervals are sufficiently small compared to the previous intervals [*α*, *β*], HybridClip is applied to line 14. Otherwise, the curve is subdivided into two subsegments and applies HybridClip to the two halves (line 12).

#### 3D curve/curve intersection

The above algorithm can be naturally generalized to handle 3D Bézier curve/curve intersection problems. In the 3D case, “fat lines” in 2D are replaced with several bounding planes, which are called “fat planes.”

Plane $\overline{L}$ passes through the end control points **P**_0_ and **P**_*n*_ of a degree *n* Bézier curve, **P**(*t*). Because a plane consists of three points that are not collinear, an arbitrary control point is simply chosen that is not on the endpoint line. Here, $\overline{L}$ is represented using the implicit equation

31 $$d\left(x,y,z\right)= ax+ by+ cz+e=0,\left({a}^2+{b}^2+{c}^2=1\right)$$

The fat plane containing curve **P**(*t*) and its control points are defined as

32 $${\mathbf{L}}_{\mathbf{P}}=\left\{\left(x,y,z\right)|d\left(x,y,z\right)\in \left[{d}_{\mathrm{min}},{d}_{\mathrm{max}}\right]\right\}$$

where [*d*_min_, *d*_max_] = [min_0 ≤ *i* ≤ *n*_*d*(**P**_*i*_), max_0 ≤ *i* ≤ *n*_*d*(**P**_*i*_)], and *d*(**P**_*i*_) = *ax*_*i*_ + *by*_*i*_ + *cz*_*i*_ + *e*, **P**_*i*_ = (*x*_*i*_, *y*_*i*_, *z*_*i*_). The distance from one curve in a cubic hybrid form is then bound to the fat plane using two cubic polynomials and a strip domain containing the intersections is computed, which is similar to Algorithm 1 described in 2D curve/curve intersection section.

## Results

### Proof for the convergence rate

Although Yuan’s method [14] is based on cubic HybClip, it is mainly used to solve univariate polynomial root problems. However, a theoretical convergence rate or proof is not provided.

In this section, the theoretical results are provided on the convergence rate of the new curve/curve intersection algorithm. This begins with two technical lemmas:

**Lemma 1. ***For any given polynomial P, there exists two constants C*_*P*_* and D*_*P*_* depending solely on P, such that for all intervals* [*α*, *β*] ⊆ [0, 1] *the lower bound m and the upper bound M generated in line 6 of Algorithm 1 satisfy*

33 $${\delta}_{\mathrm{min}}=\parallel P-m{\parallel}_{\infty}^{\left[\alpha, \beta \right]}\le {C}_P{h}^4\kern1em \mathrm{and}\kern1em {\delta}_{\mathrm{max}}=\parallel P-M{\parallel}_{\infty}^{\left[\alpha, \beta \right]}\le {D}_P{h}^4$$

where *.*

*Proof.* According to Eqs. (22) and (24), *P*(*α*) = *m*(*α*), *P*(*β*) = *m*(*β*), and *P*(*t*) ≥ *m*(*t*), ∀ *t* ∈ [*α*, *β*], and thus

34 $$P(t)-m(t)=\left(t-\alpha \right)\left(\beta -t\right)\left({P}_1(t)-{m}_1(t)\right)\ge 0$$

where *P*_1_(*t*) is a continuous function of degree *n* − 2, and *m*_1_(*t*) is a linear function. Let *g*(*t*) = *b*_0_(*β* − *t*) + *b*_1_(*t* − *α*) be a line passing through the lowest control point and parallel to the line connecting the end points of *P*_1_(*t*), such that *P*_1_(*t*) − *g*(*t*) ≥ 0, ∀ *t* ∈ [*α*, *β*], and thus

35 $${P}_1(t)-{m}_1(t)\le C\left({P}_1(t)-g(t)\right)$$

where the constant *C* depends solely on *P*.

36 $${\displaystyle \begin{array}{ll}{P}_1(t)-g(t)& =\sum\limits_{i=0}^{n-2}{a}_i{B}_{i,\left[\alpha, \beta \right]}^{n-2}(t)-\sum\limits_{i=0}^1{b}_i{B}_{i,\left[\alpha, \beta \right]}^1(t)\\ {}& =\sum\limits_{i=0}^{n-2}\left({a}_i-{c}_i\right){B}_{i,\left[\alpha, \beta \right]}^{n-2}(t),{a}_i\ge {c}_i,\forall i\\ {}& =\left(\beta -t\right){P}_2(t)+\left(t-\alpha \right){P}_3(t)\end{array}},$$

where ${\left\{{c}_i\right\}}_{i=0}^{n-2}$ are the control points of *g* after the degree elevation [1], ${P}_2(t)=\sum_{i=0}^{n-3}\left({a}_i-{c}_i\right)\left(\genfrac{}{}{0pt}{}{n-2}{i}\right){\left(\beta -t\right)}^{i-1}{\left(t-\alpha \right)}^{n-2-i}\ge 0$, and *P*_3_(*t*) = (*a*_*n* − 2_ − *c*_*n* − 2_)(*t* − *α*)^*n* − 3^ ≥ 0.

Let *t*_1_, *t*_2_ be the minimum values of *P*_2_(*t*), *P*_3_(*t*) in [*α*, *β*], respectively, i.e.,

37 $${\displaystyle \begin{array}{ll}\forall t\in \left[\alpha, \beta \right]:& {P}_2(t)\le {C}_1\left({P}_2(t)-{P}_2\left({t}_1\right)\right)={C}_1{P_2}^{\prime}\left({s}_1\right)\left(t-{t}_1\right)\le {C}_3\left(\beta -\alpha \right)\\ {}\mathrm{and}\kern1em & {P}_3(t)\le {C}_2\left({P}_3(t)-{P}_3\left({t}_2\right)\right)={C}_2{P_3}^{\prime}\left({s}_2\right)\left(t-{t}_2\right)\le {C}_4\left(\beta -\alpha \right)\end{array}}$$

where *s*_1_, *s*_2_ ∈ [*α*, *β*]. Hence,

38 $${P}_1(t)-g(t)\le {C}_3\left(\beta -t\right)\left(\beta -\alpha \right)+{C}_4\left(t-\alpha \right)\left(\beta -\alpha \right)\le {C}_5{\left(\beta -\alpha \right)}^2$$

From Eqs. (34), (35), and (38),

39 $${\displaystyle \begin{array}{ll}\left|P(t)-m(t)\right|& \le C\left(t-\alpha \right)\left(\beta -t\right)\left({P}_1(t)-g(t)\right)\\ {}& \le C\left(t-\alpha \right)\left(\beta -t\right){C}_5{\left(\beta -\alpha \right)}^2\le {C}_P{\left(\beta -\alpha \right)}^4={C}_P{h}^4\end{array}}$$

Similarly, |*M*(*t*) − *P*(*t*)| < *D*_*P*_*h*^4^.

**Lemma 2. ***For any given polynomial P, there exist constants*${C}_i^P,{D}_i^P,\mathrm{with}\ i=0,1,2,3$*depending solely on P, such that for all intervals* [*α*, *β*] ⊆ [0, 1] *the lower bound m and upper bound M generated in line 6 of Algorithm 1 for* ∀*i* ∈ {0, 1, 2, 3} *satisfy*

40 $$\parallel {P}^{(i)}-{m}^{(i)}{\parallel}_{\infty}^{\left[\alpha, \beta \right]}\le {C}_i^P{h}^{\left(4-i\right)}\kern1em \mathrm{and}\kern1em \parallel {P}^{(i)}-{M}^{(i)}{\parallel}_{\infty}^{\left[\alpha, \beta \right]}\le {D}_i^P{h}^{\left(4-i\right)}$$

*where*$$h=\beta -\alpha, \parallel r{\parallel}_{\infty}^{\left[\alpha, \beta \right]}={\max}_{t\in \left[\alpha, \beta \right]}\left|r(t)\right|$$*.*

*Proof* A new norm in [*α*, *β*] is introduced as

41 $$\parallel r{\parallel}_{\ast}^{\left[\alpha, \beta \right]}=\parallel r{\parallel}_{\infty}^{\left[\alpha, \beta \right]}+h\parallel {r}^{\prime {\parallel}_{\infty}^{\left[\alpha, \beta \right]}}+{h}^2\parallel {r}^{{\prime\prime} {\parallel}_{\infty}^{\left[\alpha, \beta \right]}}+{h}^3\parallel {r}^{(3)}{\parallel}_{\infty}^{\left[\alpha, \beta \right]}$$

According to the equivalence of norms in a finite-dimensional real linear space, there exists a constant *C* such that

42 $$\parallel r{\parallel}_{\ast}^{\left[\alpha, \beta \right]}\le C\parallel r{\parallel}_{\infty}^{\left[\alpha, \beta \right]}$$

where the constant *C* does not depend on the intervals [*α*, *β*], again owing to the affine invariance. Using arguments similar to those in the previous proof, let *r* = *P* − *m*,

43 $${\displaystyle \begin{array}{ll}& \parallel P-m{\parallel}_{\ast}^{\left[\alpha, \beta \right]}\\ {}& =\parallel P-m{\parallel}_{\infty}^{\left[\alpha, \beta \right]}+h\parallel {P}^{\prime }-{m}^{\prime }{\parallel}_{\infty}^{\left[\alpha, \beta \right]}+{h}^2\parallel {P}^{{\prime\prime} }-{m}^{{\prime\prime} }{\parallel}_{\infty}^{\left[\alpha, \beta \right]}+{h}^3\parallel {P}^{(3)}-{m}^{(3)}{\parallel}_{\infty}^{\left[\alpha, \beta \right]}\\ {}& \le C\parallel P-m{\parallel}_{\infty}^{\left[\alpha, \beta \right]}\le {C}_P{h}^4\end{array}}$$

Similarly, $$\parallel P-M{\parallel}_{\ast}^{\left[\alpha, \beta \right]}\le C\parallel P-M{\parallel}_{\infty}^{\left[\alpha, \beta \right]}\le {D}_P{h}^4$$.

From the above lemmas, the convergence rate can be analyzed using the HybClip algorithm. In Algorithm 1, if **Q** = **0**, the curve/curve intersection problem **P**(*t*) = **Q**(*s*) becomes a root-finding problem **P**(*t*) = **0**; that is, the cubic HybClip technique may be used to compute the roots of the polynomials and intersections of the two curves. These two cases are discussed separately.

**Theorem 2. ***(Single root) If polynomial P has a root t*^∗^*in* [*α*, *β*]*, and provided that this root has multiplicity 1, the sequence of the lengths of the intervals generated through cubic HybClip containing that root has the convergence rate d* = 4*.*

*Proof.* Suppose that ([*α*_*i*_, *β*_*i*_])_*i* = 0, 1, 2, …_, which converges to *t*^∗^, is a sequence of intervals generated by Algorithm 1, with lengths *h*_*i*_ = *β*_*i*_ − *α*_*i*_. It is assumed that the first derivative satisfies *P*^′^(*t*^∗^) > 0 (otherwise, the polynomial −*P* can be considered instead of *P*).

Two cubic Bernstein polynomials *m* and *M* can be obtained as the lower and upper bounds of *P* in [*α*_*i*_, *β*_*i*_] based on line 6 of Algorithm 1. Because *P*^′^ is continuous, and owing to Lemma 2, the following inequalities

44 $${\displaystyle \begin{array}{ll}& \parallel {P}^{\prime }-{P}^{\prime}\left({t}^{\ast}\right){\parallel}_{\infty}^{\left[{\alpha}_i,{\beta}_i\right]}\le \frac{1}{4}{P}^{\prime}\left({t}^{\ast}\right)\kern1em \mathrm{and}\kern1em \parallel {m}^{\prime }-{P}^{\prime }(t){\parallel}_{\infty}^{\left[{\alpha}_i,{\beta}_i\right]}\le \frac{1}{4}{P}^{\prime}\left({t}^{\ast}\right)\\ {}& \parallel {M}^{\prime }-{P}^{\prime }(t){\parallel}_{\infty}^{\left[{\alpha}_i,{\beta}_i\right]}\le \frac{1}{4}{P}^{\prime}\left({t}^{\ast}\right)\end{array}}$$

hold for all but a finite number of values of *i*. These three inequalities above imply that

45 $${\displaystyle \begin{array}{ll}& \parallel {m}^{\prime }-{P}^{\prime}\left({t}^{\ast}\right){\parallel}_{\infty}^{\left[{\alpha}_i,{\beta}_i\right]}\le \parallel {P}^{\prime }-{P}^{\prime}\left({t}^{\ast}\right){\parallel}_{\infty}^{\left[{\alpha}_i,{\beta}_i\right]}+\parallel {m}^{\prime }-{P}^{\prime }{\parallel}_{\infty}^{\left[{\alpha}_i,{\beta}_i\right]}\le \frac{1}{2}{P}^{\prime}\left({t}^{\ast}\right)\\ {}& \parallel {M}^{\prime }-{P}^{\prime}\left({t}^{\ast}\right){\parallel}_{\infty}^{\left[{\alpha}_i,{\beta}_i\right]}\le \parallel {P}^{\prime }-{P}^{\prime}\left({t}^{\ast}\right){\parallel}_{\infty}^{\left[{\alpha}_i,{\beta}_i\right]}+\parallel {M}^{\prime }-{P}^{\prime }{\parallel}_{\infty}^{\left[{\alpha}_i,{\beta}_i\right]}\le \frac{1}{2}{P}^{\prime}\left({t}^{\ast}\right)\end{array}}$$

and hence

46 $$\forall t\in \left[{\alpha}_i,{\beta}_i\right]:{m}^{\prime (t)}\ge \frac{1}{2}{P}^{\prime \left({t}^{\ast}\right)},{M}^{\prime (t)}\ge \frac{1}{2}{P}^{\prime \left({t}^{\ast}\right)}$$

**Fig. 4:**
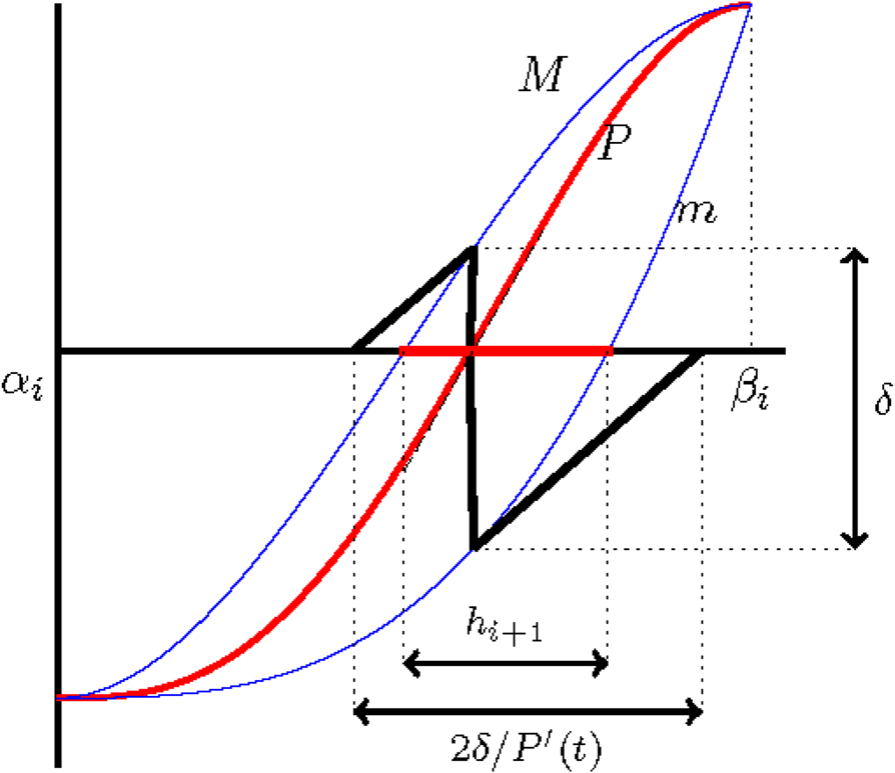
Illustration of Theorem 2

From Lemma 1, the vertical height *δ* = *δ*_min_ + *δ*_max_ of *m* and *M* is bounded by ${C}_P{h}_i^4$. Thus, the length *h*_*i*_ of the intervals satisfies

47 $${h}_{i+1}\le \frac{2\delta }{P^{\prime \left({t}^{\ast}\right)}}\le \frac{2{C}_P}{P^{\prime \left({t}^{\ast}\right)}}{h}_i^4$$

for all but a finite number of values of *i* (Fig. 4).

**Fig. 5:**
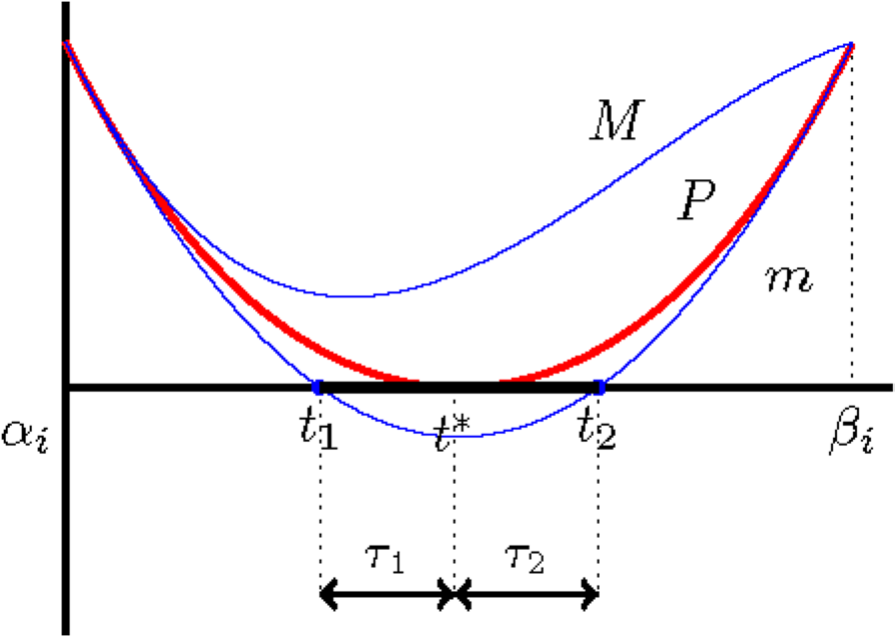
Illustration of Theorem 3

For other clipping techniques [8, 9], multiple roots reduce the convergence rate. The convergence rate of cubic HybClip is now discussed in the case of double roots, as illustrated in Fig. 5.

**Theorem 3. ***(Double root) If the polynomial P has a root t*^∗^*in* [*α*, *β*]*, and provided that this root has multiplicity 2, the sequence of the lengths of the intervals generated by cubic HybClip containing that root has the convergence rate d* = 2*.*

*Proof* Similar to the proof of the previous theorem, the sequence of intervals ([*α*_*i*_, *β*_*i*_])_*i* = 0, 1, 2, …_ is analyzed with lengths *h*_*i*_ = *β*_*i*_ − *α*_*i*_ generated by Algorithm 1, which contains the double root. It is assumed that the second derivative satisfies *P*^″^ > 0. Otherwise, polynomial −*P* can be considered instead of *P*.

Again, two cubic Bernstein polynomials *m* and *M* can be obtained as the lower and upper bounds of *P* in [*α*_*i*_, *β*_*i*_]. Because *P*^″^ is continuous, and based on Lemma 2, the inequalities

48 $$\parallel {P}^{{\prime\prime} }-{P}^{{\prime\prime}}\left({t}^{\ast}\right){\parallel}_{\infty}^{\left[{\alpha}_i,{\beta}_i\right]}\le \frac{1}{4}{P}^{{\prime\prime}}\left({t}^{\ast}\right)\kern1em \mathrm{and}\kern1em \parallel {m}^{{\prime\prime} }-{P}^{{\prime\prime} }(t){\parallel}_{\infty}^{\left[{\alpha}_i,{\beta}_i\right]}\le \frac{1}{4}{P}^{{\prime\prime}}\left({t}^{\ast}\right)$$

hold for all but a finite number of values of *i*. These two inequalities imply that

49 $$\parallel {m}^{{\prime\prime} }-{P}^{{\prime\prime} \left({t}^{\ast}\right){\parallel}_{\infty}^{\left[{\alpha}_i,{\beta}_i\right]}}\le \parallel {P}^{{\prime\prime} }-{P}^{{\prime\prime} \left({t}^{\ast}\right){\parallel}_{\infty}^{\left[{\alpha}_i,{\beta}_i\right]}}+\parallel {m}^{{\prime\prime} }-{P}^{{\prime\prime} {\parallel}_{\infty}^{\left[{\alpha}_i,{\beta}_i\right]}}\le \frac{1}{2}{P}^{{\prime\prime} \left({t}^{\ast}\right)}$$

and thus ${m}^{{\prime\prime} }(t)\ge \frac{1}{2}{P}^{{\prime\prime}}\left({t}^{\ast}\right),\forall t\in \left[{\alpha}_i,{\beta}_i\right]$. Letting *τ* = *t* − *t*^∗^, and based on

50 $$\overset\leftharpoonup m\left(\tau\right)=m(t)=b_3\tau^3+b_2\tau^2+b_1\tau+b_0,b_i=\frac1{i!}m^{(i)}\left(t^\ast\right)$$

$\left|{b}_2\right|=\frac{1}{2}{m}^{{\prime\prime}}\left({t}^{\ast}\right)\ge \frac{1}{4}{P}^{{\prime\prime}}\left({t}^{\ast}\right)>0$. From Lemmas 1 and 2,

51 $${\displaystyle \begin{array}{ll}\left|{b}_0\right|& =\left|m\left({t}^{\ast}\right)\right|=\left|m\left({t}^{\ast}\right)-P\left({t}^{\ast}\right)\right|\le {C}_{0P}{h}_i^4\\ {}\left|{b}_1\right|& =\left|{m}^{\prime}\left({t}^{\ast}\right)\right|=\left|{m}^{\prime}\left({t}^{\ast}\right)-{P}^{\prime}\left({t}^{\ast}\right)\right|\le {C}_{1P}{h}_i^3\\ {}\left|{b}_3\right|& =\left|\frac{1}{6}{m}^{(3)}\left({t}^{\ast}\right)\right|\le \frac{1}{6}\left|{P}^{(3)}\left({t}^{\ast}\right)\right|+\frac{1}{6}\left|{m}^{(3)}\left({t}^{\ast}\right)-{P}^{(3)}\left({t}^{\ast}\right)\right|\\ {}& \le \frac{1}{6}\left|{P}^{(3)}\left({t}^{\ast}\right)\right|+\frac{1}{6}{C}_{3P}{h}_i:= {D}_{3P}\end{array}}$$

Letting *t*_1_, *t*_2_ be the roots of *m*, *t*^∗^ ∈ [*t*_1_, *t*_2_], and *τ*_2_ = *t*_2_ − *t*^∗^ > 0, *τ*_1_ = *t*_1_ − *t*^∗^ < 0, the following is obtained:

52 $${\displaystyle \begin{array}{ll}\left|{b}_2{\tau}_1^2\right|& \le \left|{b}_3{\tau}_1^3\right|+\left|{b}_1{\tau}_1\right|+\left|{b}_0\right|\\ {}& \le {\tau}_1^2\cdot {D}_{3P}\left|{\tau}_1\right|+{C}_{1P}{h}_i^4+{C}_{0P}{h}_i^4:= {\tau}_1^2\cdot {D}_{3P}\left|{\tau}_1\right|+{D}_{2P}{h}_i^4\end{array}}$$

Because *τ*_1_ ≤ *h*_*i*_ and *h*_*i*_ → 0, *D*_3*P*_|*τ*_1_| → 0,

53 $$\left|{b}_2{\tau}_1^2\right|\le \frac{1}{2}\left|{b}_2\right|\left|{\tau}_1^2\right|+{D}_{2P}{h}_i^4$$

for a sufficiently large *i*. Therefore, ${D}_{2P}{h}_i^4\ge \frac{1}{2}\left|{b}_2\right|\left|{\tau}_1^2\right|\ge \frac{1}{8}{P}^{{\prime\prime}}\left({t}^{\ast}\right)\left|{\tau}_1^2\right|$, and hence

54 $${\tau}_1\le {\left(\frac{8{D}_{2P}}{P^{{\prime\prime} \left({t}^{\ast}\right)}}\right)}^{\frac{1}{2}}{h}_i^2$$

Similarly, the following bound for *t*_2_ is obtained:

55 $${\tau}_2\le {\left(\frac{8D{\prime}_{2P}}{P^{{\prime\prime} \left({t}^{\ast}\right)}}\right)}^{\frac{1}{2}}{h}_i^2$$

Because *τ*_1_ < 0, *τ*_2_ > 0,

56 $${h}_{i+1}=\left|{t}_2-{t}_1\right|={\tau}_2-{\tau}_1\le \left({\left(\frac{8{D}_{2P}}{P^{{\prime\prime} \left({t}^{\ast}\right)}}\right)}^{\frac{1}{2}}-{\left(\frac{8D{\prime}_{2P}}{P^{{\prime\prime} \left({t}^{\ast}\right)}}\right)}^{\frac{1}{2}}\right){h}_i^2$$

Hence, the sequence (*h*_*i*_)_*i* = 0, 1, 2, …_ has a convergence rate of 2.

From Theorems 2 and 3, it can be seen that the new algorithm has a higher convergence rate compared with geometry interval clipping [11] and quadratic clipping [8] when computing all roots of a univariate polynomial equation. The following theorem provides the convergence rate for the curve/curve intersection problems.

**Theorem 4. ***Suppose ***f**(*t*), **g**(*s*) *have a transversal intersection* (**f**^′^(*t*^∗^) × **g**^′^(*s*^∗^) ≠ 0) *at ***p**^∗^ = **f**(*t*^∗^) = **g**(*s*^∗^)*. Furthermore, supposing that* [*α*_*i*_, *β*_*i*_]_*i* = 0, 1, 2, …_*is the sequence of generated intervals that contain t*^∗^*, and* [*ξ*_*i*_, *η*_*i*_]_*i* = 0, 1, 2, …_*is the corresponding sequence of generated intervals that contain s*^∗^*, there then exist constants C*_1_*, C*_2_*, C*_3_*, C*_4_ *depending solely on ***f ***and ***g***, such that*

57 $${h}_{i+1,\mathbf{f}}\le {C}_1{h}_{i,\mathbf{f}}^4+{C}_2{h}_{i,\mathbf{g}}^2\kern1em \mathrm{and}\kern1em {h}_{i+1,\mathbf{g}}\le {C}_3{h}_{i,\mathbf{g}}^4+{C}_4{h}_{i,\mathbf{f}}^4$$

*Proof* From line 11 of Algorithm 1, it can be seen that the length of intervals [*ξ*_*i*_, *η*_*i*_] tends toward zero as *i* tends toward infinity, that is, the interval [*ξ*_*i*_, *η*_*i*_] tends toward *s*^∗^.

Let ${\overline{L}}_{\mathbf{g}}$ be the line or plane that passes through the endpoints **b**_0_, **b**_*m*_ of **g** in [*ξ*_*i*_, *η*_*i*_]. Denote **n** as the unit normal vector of ${\overline{L}}_{\mathbf{g}}$. Then, the distance function from **f**(*t*) to ${\overline{L}}_{\mathbf{g}}$ is defined as

58 $$d(t)=\mathbf{n}\cdot \left(\mathbf{f}(t)-{\mathbf{b}}_0\right)$$

Denote ${\mathbf{T}}_{\mathbf{f}}^{\ast }$ as the tangent line of **f** at *t*^∗^. Let $\phi \in \left[0,\frac{\pi }{2}\right]$ be the angle between ${\mathbf{T}}_{\mathbf{f}}^{\ast }$ and ${\overline{L}}_{\mathbf{g}}$, and $\theta \in \left[0,\frac{\pi }{2}\right]$ be the angles between ${\mathbf{T}}_{\mathbf{f}}^{\ast }$ and **b**_0_**b**_*m*_. As *h*_*i*, **g**_ = [*ξ*_*i*_, *η*_*i*_] tends toward 0, the line or plane ${\overline{L}}_{\mathbf{g}}$ converges at **b**_0_**b**_*m*_, and angle *φ* converges at *θ*. Thus, for a sufficiently small *h*_*i*, **g**_, $\phi >\frac{\theta }{2}>0$, and thus $0<\sin \left(\frac{\theta }{2}\right)<\sin \left(\phi \right)\le 1$.

The angle *ρ* between **f**^′^(*t*^∗^) and **n** is either $\rho =\frac{\pi }{2}+\phi$ or $\rho =\frac{\pi }{2}-\phi$. Using this, the derivative of the distance function can be bound at the intersection as

59 $$\left|{d}^{\prime}\left({t}^{\ast}\right)\right|=\left|\mathbf{n}\cdot {\mathbf{f}}^{\prime}\left({t}^{\ast}\right)\right|=\parallel {\mathbf{f}}^{\prime}\left({t}^{\ast}\right)\parallel \left|\cos \left(\frac{\pi }{2}\pm \phi \right)\right|=\parallel {\mathbf{f}}^{\prime}\left({t}^{\ast}\right)\parallel \sin \left(\phi \right)>0$$

Because *d*^′^(*t*^∗^) ≠ 0, and for convenience, *w* = *d*^′^(*t*^∗^) > 0 is denoted (otherwise, the vector −**n** can be considered instead of **n**).

Because *d*^′^(*t*) is continuous, the inequality

60 $$\parallel {d}^{\prime }-{d}^{\prime \left({t}^{\ast}\right){\parallel}_{\infty}^{\left[{\alpha}_i,{\beta}_i\right]}}<\frac{w}{2}$$

holds for all but a finite number of values of *i*. Hence,

61 $$\forall t\in \left[{\alpha}_i,{\beta}_i\right],{d}^{\prime (t)}>\frac{w}{2}$$

From line 6 of Algorithm 1, the cubic polynomial bound [*d*_*m*_(*t*), *d*_*M*_(*t*)] of the distance function *d*(*t*) can be obtained. Based on Lemma 2,

62 $$\parallel {d}^{\prime }-d{\prime}_m{\parallel}_{\infty}^{\left[{\alpha}_i,{\beta}_i\right]}\le \frac{w}{4}\kern1em \mathrm{and}\kern1em \parallel {d}^{\prime (t)}-d{\prime}_M(t){\parallel}_{\infty}^{\left[{\alpha}_i,{\beta}_i\right]}\le \frac{w}{4}$$

and by Eq. (61), the following is obtained:

63 $$d{\prime}_m(t)\ge \frac{w}{4}\kern1em \mathrm{and}\kern1em d{\prime}_M(t)\ge \frac{w}{4}$$

**Fig. 6:**
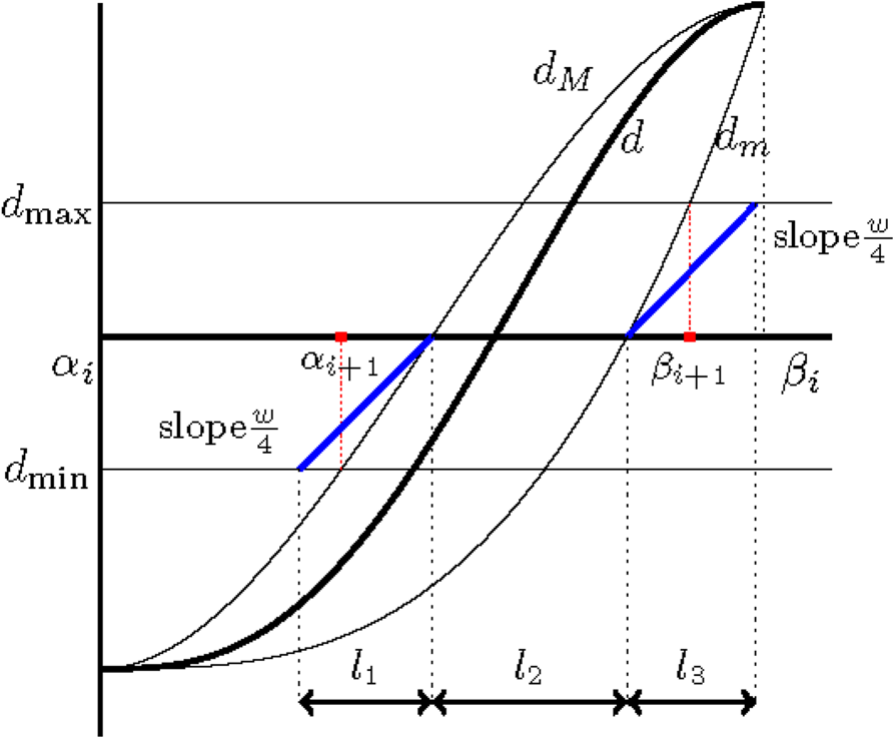
Illustration of Eq. (64)

From Fig. 6, the bound for *h*_*i* + 1, **f**_ is obtained as

64 $${\displaystyle \begin{array}{ll}& {h}_{i+1,\mathbf{f}}={\beta}_{i+1}-{\alpha}_{i+1}\le {l}_1+{l}_2+{l}_3\\ {}& {l}_1+{l}_3=\frac{d_{\mathrm{max}}-{d}_{\mathrm{min}}}{w/4}\end{array}}$$

Based on Lemma 1, the vertical heights *δ*_*i*_ of *d*_*m*_ and *d*_*M*_ are bounded as follows:

65 $${\delta}_i=\parallel {d}_M-{d}_m{\parallel}_{\infty}^{\left[{\alpha}_i,{\beta}_i\right]}\le \parallel {d}_M-d{\parallel}_{\infty}^{\left[{\alpha}_i,{\beta}_i\right]}+\parallel d-{d}_m{\parallel}_{\infty}^{\left[{\alpha}_i,{\beta}_i\right]}\le {C}_{\mathbf{f}}{h}_i^4$$

Let *t*_1_ and *t*_2_ be the roots of *d*_*m*_ and *d*_*M*_ respectively. From Eq. (63),

66 $${l}_2=\left|{t}_1-{t}_2\right|<\frac{\delta_i}{w/4}=\frac{C_{\mathbf{f}}{h}_i^4}{w/4}$$

From Eq. (64), the above inequality implies that

67 $${h}_{i+1,\mathbf{f}}<\frac{d_{\mathrm{max}}-{d}_{\mathrm{min}}}{w/4}+\frac{C_{\mathbf{f}}{h}_i^4}{w/4}$$

This thus implies the first inequality in Eq. (57) from ${d}_{\mathrm{max}}-{d}_{\mathrm{min}}<{h}_{i,\mathbf{g}}^2{C}_{\mathbf{g}}$ (see the proof of Theorem 6 in ref. [7]). Similarly, in the next iteration step, the following is obtained:

68 $${h}_{i+1,\mathbf{g}}\le C{\prime}_{\mathbf{g}}{h}_{i,\mathbf{g}}^4+C{\prime}_{\mathbf{f}}{h}_{i+1,\mathbf{f}}^2$$

where *C*′_**f**_ is solely dependent on **f**, and *C*′_**g**_ is solely dependent on **g**. Based on the first inequality of Eq. (57), the following is obtained:

69 $${\displaystyle \begin{array}{ll}{h}_{i+1,\mathbf{g}}& \le C{\prime}_{\mathbf{g}}{h}_{i,\mathbf{g}}^4+C{\prime}_{\mathbf{f}}{\left({C}_{\mathbf{f}}{h}_{i,\mathbf{f}}^4+{C}_{\mathbf{g}}{h}_{i,\mathbf{g}}^2\right)}^2\\ {}& \le C{\prime}_{\mathbf{g}}{h}_{i,\mathbf{g}}^4+C{\prime}_{\mathbf{f}}\left({C}_{\mathbf{f}}^2{h}_{i,\mathbf{f}}^8+{C}_{\mathbf{g}}^2{h}_{i,\mathbf{g}}^4+2{C}_{\mathbf{f}}{C}_{\mathbf{g}}{h}_{i,\mathbf{f}}^4{h}_{i,\mathbf{g}}^2\right)\\ {}& \le \left(C{\prime}_{\mathbf{g}}+C{\prime}_{\mathbf{f}}{C}_{\mathbf{g}}^2\right){h}_{i,\mathbf{g}}^4+\left(C{\prime}_{\mathbf{f}}{C}_{\mathbf{f}}^2{h}_{i,\mathbf{f}}^4+2C{\prime}_{\mathbf{f}}{C}_{\mathbf{f}}{C}_{\mathbf{g}}{h}_{i,\mathbf{g}}^2\right){h}_{i,\mathbf{f}}^4\end{array}}$$

which implies the second inequality.

Note that the property of *w* being nonzero is key to binding *l*_1_ and *l*_3_. Therefore, a transversal intersection is required in the proof. From Theorem 4, the two sequences {[*α*_*i*_, *β*_*i*_]}_*i*_ and {[*ξ*_*i*_, *η*_*i*_]}_*i*_ of the new intersection algorithm have second- and fourth-order convergence rates, respectively, and the 3D curve intersection problem yields the same results.

### Experimental results

In this section, all six algorithms are compared based on three criteria: the amount of time per iteration step, the number of iterations, and the computing time required to achieve a certain accuracy. All algorithms were implemented in C++ on a PC with an 2.60-GHz Intel^(R)^ Core^(TM)^ i7-9750H CPU and 16.0 GB of RAM. In all experiments, both curves **P**(*t*) and **Q**(*s*) have a parameter domain [0, 1].

For convenience, denote Bézier clipping as BezClip [5]; quadratic clipping and cubic clipping based on a degree reduction as 2-DegClip [8] and 3-DegClip [10], respectively; geometry interval clipping as 2-HybClip [11]; and cubic HybClip based on hybrid curves in ref. [14] as 3-HybClip*. In addition, the proposed cubic HybClip algorithm is denoted as 3-HybClip.

**Fig. 7:**
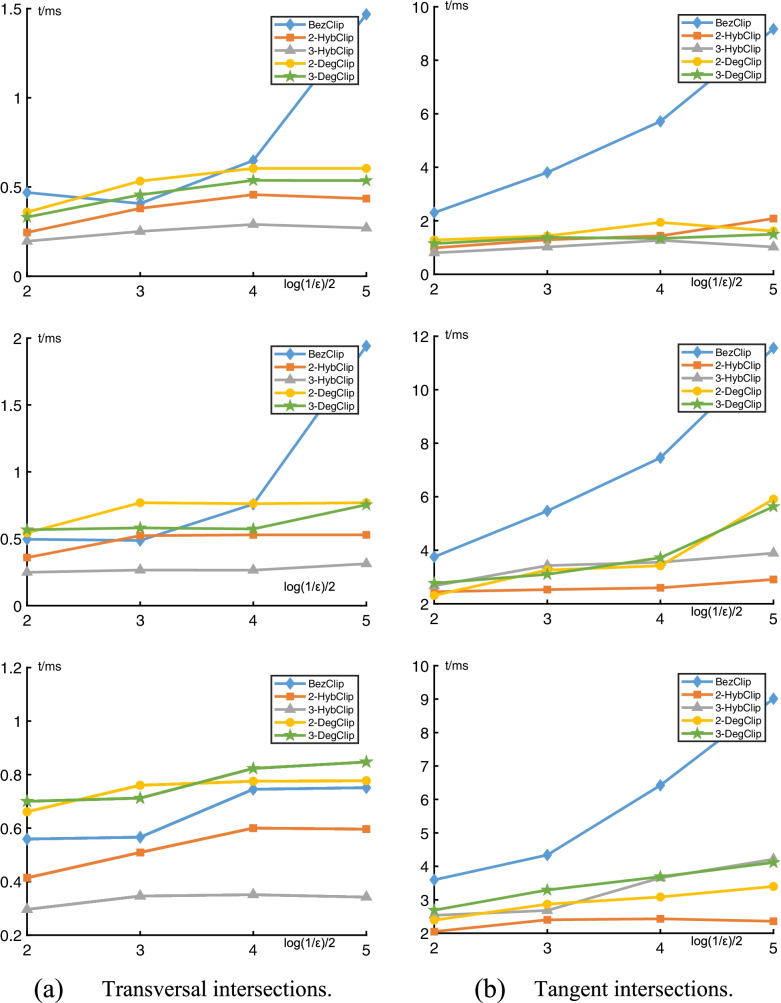
Computing time *t* in milliseconds vs accuracy *ε* of curve pairs with degrees (4, 4), (8, 4), and (8, 8) from the top to down. **a** Transversal intersections; **b** Tangent intersections

To analyze the relationship between the computational effort and the desired accuracy, two examples representing polynomials with transversal and tangent intersections are discussed. The five algorithms are first applied to three pairs of Bézier curves with a transversal intersection. Table 1 reports the number of pairs of iterations and the computing time in microseconds of the desired accuracy for computing the transversal intersections between the three curve pairs with various degrees. Figure **7**a shows the relationship between the computing time and desired accuracy, and indicates that 3-HybClip based on cubic hybrid curves is significantly improved in comparison with BezClip, 2HybClip, 2-DegClip, and 3-DegClip.

70 $$\left\{\begin{array}{c}{\mathbf{P}}_4(t)=\left(\left(t-1/2\right)\left(t-3\right){\left(t+1\right)}^2,\left(t-1/2\right)\left(t-2\right){\left(t+1\right)}^2\right)\\ {}{\mathbf{Q}}_4(s)=\left(\left(s-1/2\right)\left(s-2\right){\left(s+2\right)}^2,\left(s-1/2\right){\left(s-2\right)}^2\left(s+1\right)\right)\end{array}\right.$$

71 $$\left\{\begin{array}{c}{\mathbf{P}}_8(t)=\left(\left(t-1/2\right){\left(t-2\right)}^4{\left(t+1/2\right)}^3,\left(t-1/2\right){\left(t-2\right)}^4{\left(t+1\right)}^3\right)\\ {}{\mathbf{Q}}_4(s)=\left(\left(s-1/2\right)\left(s-2\right){\left(s+2\right)}^2,\left(s-1/2\right){\left(s-2\right)}^2\left(s+1\right)\right)\end{array}\right.$$

72 $$\left\{\begin{array}{c}{\mathbf{P}}_8(t)=\left(\left(t-1/2\right){\left(t-2\right)}^4{\left(t+1/2\right)}^3,\left(t-1/2\right){\left(t-2\right)}^4{\left(t+1\right)}^3\right)\\ {}{\mathbf{Q}}_8(s)=\left(\left(s-1/2\right){\left(s-1\right)}^3{\left(s+1\right)}^4,\left(s-1/2\right){\left(s-2\right)}^4{\left(s+1\right)}^3\right)\end{array}\right.$$

**Table 1:** Transversal intersections

(*n*, *m*)	*ε*	10^−6^					10^−10^				
		BezClip	2-HybClip	3-HybClip	2-DegClip	3-DegClip	BezClip	2-HybClip	3-HybClip	2-DegClip	3-DegClip
(4,4)	[*N*_**P**_, *N*_**Q**_]	[4,3]	[3,3]	[3,2]	[3,3]	[3,2]	[16,15]	[4,3]	[3,3]	[4,3]	[3,3]
	*t*/*μs*	125	120	120	150	150	90	90	100	125	130
(8,4)	[*N*_**P**_, *N*_**Q**_]	[4,3]	[3,3]	[3,2]	[3,3]	[3,2]	[15,14]	[4,3]	[3,2]	[4,3]	[3,3]
	*t*/*μs*	160	150	150	190	190	100	120	100	140	130
(8,8)	[*N*_**P**_, *N*_**Q**_]	[4,4]	[4,4]	[3,3]	[3,3]	[3,3]	[16,16]	[5,4]	[3,3]	[4,4]	[3,3]
	*t*/*μs*	190	180	180	250	250	190	130	140	180	170

Number of iterations [*N*_***P***_, *N*_***Q***_] and computing time *t* (in microseconds) of accuracy *ε*. In addition, (*n*, *m*) are degrees of ***P***, ***Q***, respectively

The five algorithms are applied to three pairs of Bézier curves with tangent intersections. Table 2 and Fig. 7b report the number of pairs of iterations and the computing time in milliseconds of various desired accuracies *ε* for computing the tangent intersections between the three curve pairs with various degrees. Experimental results show that the quadratic and cubic clipping techniques are better than Bézier clipping; however, compared with quadratic clipping based on hybrid curves or a degree reduction, the cubic clipping techniques show no substantial improvements. This is due to the fact that all clipping algorithms require a large number of binary subdivisions for tangent intersections.

73 $$\left\{\begin{array}{c}{\mathbf{P}}_4(t)=\left(2t-1,-4{t}^4+8{t}^3-4t+1.5\right)\\ {}{\mathbf{Q}}_4(s)=\left(2s-1,4{s}^4-8{s}^3+4s-1\right)\end{array}\right.$$

74 $$\left\{\begin{array}{c}{\mathbf{P}}_8(t)=\left(2t-1,-20{t}^8+80{t}^7-112{t}^6+56{t}^5-4t+1.7031\right)\\ {}{\mathbf{Q}}_4(s)=\left(2s-1,4{s}^4-8{s}^3+4s-1\right)\end{array}\right.$$

75 $$\left\{\begin{array}{c}{\mathbf{P}}_8(t)=\left(2t-1,-20{t}^8+80{t}^7-112{t}^6+56{t}^5-4t+1.7031\right)\\ {}{\mathbf{Q}}_8(s)=\left(2s-1,20{s}^8-80{s}^7+112{s}^6-56{s}^5+4s-1.2031\right)\end{array}\right.$$

**Table 2:** Tangent intersections

(*n*, *m*)	*ε*	10^−6^					10^−10^				
		BezClip	2-HybClip	3-HybClip	2-DegClip	3-DegClip	BezClip	2-HybClip	3-HybClip	2-DegClip	3-DegClip
(4,4)	[*N*_**P**_, *N*_**Q**_]	[12,10]	[6,5]	[6,5]	[6,6]	[5,5]	[20,20]	[6,6]	[6,6]	[7,6]	[6,5]
	*t*/*μs*	138	117	93	119	138	164	129	85	124	136
(8,4)	[*N*_**P**_, *N*_**Q**_]	[20,20]	[8,8]	[10,10]	[6,6]	[5,6]	[34,34]	[9,8]	[11,10]	[7,6]	[5,7]
	*t*/*μs*	160	175	170	190	200	160	175	185	232	246
(8,8)	[*N*_**P**_, *N*_**Q**_]	[18,17]	[7,7]	[10,9]	[7,7]	[4,6]	[32,30]	[8,7]	[10,10]	[8,7]	[5,6]
	*t*/*μs*	170	185	180	226	263	177	157	133	272	282

Number of iterations [*N*_***P***_, *N*_***Q***_] and computing time *t* (in microseconds) of accuracy *ε*. In addition, (*n*, *m*) are degrees of ***P***, ***Q***, respectively

**Fig. 8:**
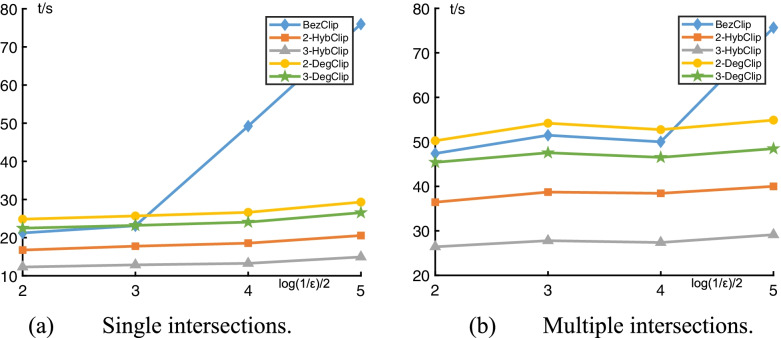
Statistical comparisons: Computing time *t* in seconds vs accuracy *ε*. **a** Single intersections; **b** Multiple intersections

To compare these six algorithms numerically, statistics are generated on 40,000 pairs of randomly generated polynomial curves of degree 4–10 for single and multiple intersections. Figure 8 shows computing time needed to achieve the given accuracy of the five algorithms. The relative computing iterations and computing time for these tests are listed in Table 3. As shown in Table 3, 3-HybClip requires 2% fewer iterations and 8% less time than 3-HybClip* [14]. In addition, 3-HybClip has 2% fewer computing iterations than 3-DegClip, and at least 10% fewer iterations than 2-HybClip and 2-DegClip. With respect to the computing time, 3-HybClip is at least 60% faster than 3-DegClip and 2-DegClip, and at least 30% faster than 2-HybClip.

**Table 3:** Relative computing iterations *N* and computing time *t*

ε		BezClip	2-HybClip	3-HybClip*	3-HybClip	2-DegClip	3-DegClip
10^−6^	*N*	1.36	1.14	1.02	1	1.18	1.02
	*t*	1.85	1.39	1.08	1	1.95	1.71
10^−10^	*N*	1.79	1.16	1.03	1	1.18	1.03
	*t*	2.60	1.37	1.09	1	1.88	1.66

## Conclusions

In this study, an algorithm called 3-HybClip was derived for computing all intersections between two Bézier curves within a given domain. By selecting the moving control points, better bounds were obtained than those in ref. [14]. It was proved that the two sequences of bounded intervals for intersections have second- and fourth-order convergence rates for transversal intersections. The experimental results show that the newly proposed 3-HybClip algorithm requires 2% fewer iterations and 8% less time than 3-HybClip* from ref. [14], 10% fewer iterations than 2-HybClip and 2-DegClip, and at least 30% less time than other techniques such as BezClip, 2-HybClip, 2-DegClip, and 3-DegClip.

## Discussion

As discussed in 3D curve/curve intersection section, for 3D curve/curve intersection problems, the “fat planes” are computed to bound a 3D Bézier curve. The distance from one curve in a cubic hybrid form to the fat plane is bound by two cubic polynomials, and a strip domain containing the intersections is then computed. Similarly, in curve/surface intersection problems, “fat planes” can also be used to bind a Bézier surface, and then the distance from the curve to the fat plane is bound by two cubic polynomials. Then the intersection of “fat planes” and the two cubic polynomials is the strip domain containing the intersections. The details of the algorithm and comparisons with previous approaches are left for future work.

## Data Availability

Code sets and date sets: https://gitee.com/yaqiong-wu/CurveCurveIntersect.git.

## Abbreviations

CAD/CAM: Computer-aided design and manufacturing
HybClip: Hybrid clipping
